# ‘Sustainable Minds’: Examining Clinician Engagement in Sustainable Mental Health Care Practices at Bradford District Care NHS Foundation Trust

**DOI:** 10.1192/bjo.2024.362

**Published:** 2024-08-01

**Authors:** Sana Fatima, Deepak Moyal

**Affiliations:** BDCT, Bradford, United Kingdom

## Abstract

**Aims:**

The burgeoning focus on climate change has emerged as a prominent area of both interest and concern. As the importance of sustainable healthcare practices gains momentum, there is a heightened focus on tackling environmental issues and promoting planetary health. In a noteworthy achievement, the NHS became the world's first health service in October 2020 to commit to achieving carbon net zero.

Bradford District Care NHS Foundation Trust (BDCT) has been a strong advocate for sustainability and planetary health (S&PH) initiatives, driven by the committed leadership of the BDCT Sustainability department. While the non-clinical senior leadership displayed active involvement in these initiatives, there appeared to be, at least anecdotally, a somewhat limited participation from the clinical teams. This project aimed to investigate and analyse this perceived gap, utilising the findings to guide future initiatives.

*Aims and Objectives:*

The primary objectives of this research are to assess, evaluate, and empower medical staff at BDCT in sustainable healthcare practices. The specific aims include:

1. Assessing the current awareness levels of S&PH among medical staff.
2. Evaluating the extent of medical staff involvement in existing S&PH initiatives within the NHS and BDCT.
3. Identifying barriers and challenges faced by medical staff in actively participating in sustainability and environmental initiatives.
4. Contributing insights to broader S&PH initiatives within BDCT.
5. Developing strategies to empower clinicians, service users, and communities to actively engage in environmental and sustainability initiatives.

Drawing inspiration from the Royal College of Psychiatrists (RCPsych), a champion of S&PH, this project centres on a broader perspective that: sustainability extends beyond carbon counting and includes not only climate and environmental initiatives, but also considers sustainability of the workforce as firmly within the remit of sustainability.

We focused on three key themes: the staff, the patients, and the access to green spaces.

**Methods:**

A research tool was developed to conduct this project. This entailed an online, semi structured proforma which was disseminated across the medical staff group in BDCT. The medical staff group included consultants, trainees, SAS/Trust Grade, and LAS doctors.

The proforma consisted of 18 questions and examined the following three themes:

- Staff: Staff Knowledge and Awareness of S&PH
- Patients: How do staff facilitate patients' awareness of S&PH?
- WorkPlace: Access to green spaces.

The collected data was analysed to derive insights, which were formulated to inform our action plan.

**Results:**

Total 18 responses gathered via the semi structured proforma. 55% (n = 10) responses were from inpatient setting, 33.3% (n = 6) from community setting; 2 from other settings. 72.2% (n = 13) were in full time occupation while rest (n = 5) were less than full time.

Genders had almost equal representation.

Only a minority (27%) of staff were aware of the sustainability champion within BDCT, while only 22.2% were aware about the BDCT Green Plan initiative.

The common suggestions about methods to improve the awareness about the Green Plan and Sustainability were email, intranet page, sessions on the topic, posters, monthly update amongst various others. Email newsletter was overrepresented as a common theme (24%).

A majority of employees (78%) noted a significant change in the mileage which was a reduction (78% noted a reduction) as expected before and after Covid Pandemic.

Some of the suggestions received were innovative and interesting including cycle scheme, working from home, hybrid working amongst others. However, almost all responses were able to appraise the advantages of flexible working and the associated barriers.

The last segment explored the green spaces and their effect on mental health which was very encouraging.

**Conclusion:**

This research project aligns with the RCPsych's vision of sustainability and nature, emphasising the critical role clinicians play in advancing sustainable healthcare practices. Some of the findings are really encouraging, however there is a still a significant gap in understanding the role of sustainability champion and the Green Plan.

By exploring awareness levels, involvement, and challenges faced by medical staff, this study seeks to provide actionable insights that not only enhance practices within BDCT but also contribute to the broader discourse on sustainability and planetary health in healthcare settings.

The findings are anticipated to catalyse strategies that empower clinicians and communities, fostering a culture of active engagement in environmental and sustainability initiatives.

